# Environmental identification of arbuscular mycorrhizal fungi using the LSU rDNA gene region: an expanded database and improved pipeline

**DOI:** 10.1007/s00572-022-01068-3

**Published:** 2022-01-31

**Authors:** Camille S. Delavaux, Robert J. Ramos, Sidney L. Sturmer, James D. Bever

**Affiliations:** 1 Department of Ecology and Evolutionary Biology, The University of Kansas, 2041 Haworth Hall, 1200 Sunnyside Avenue, Lawrence, KS 66045 USA; 2 Kansas Biological Survey, The University of Kansas, 106 Higuchi Hall, 2101 Constant Ave, Lawrence, KS 66047 USA; 3 Department of Environmental Systems Science, ETH Zurich, Universitätstrasse 16, 8092 Zurich, Switzerland; 4 Departamento de Ciências Naturais, Universidade Regional de Blumenau, R. Antônio da Veiga 140Santa Catarina, Blumenau, 89030-903 Brazil

**Keywords:** Glomeromycota, Amplicon sequencing, Large subunit, Environmental DNA, Phylogenetics, Bioinformatics

## Abstract

Arbuscular mycorrhizal fungi (AMF; Glomeromycota) are difficult to culture; therefore, establishing a robust amplicon-based approach to taxa identification is imperative to describe AMF diversity. Further, due to low and biased sampling of AMF taxa, molecular databases do not represent the breadth of AMF diversity, making database matching approaches suboptimal. Therefore, a full description of AMF diversity requires a tool to determine sequence-based placement in the Glomeromycota clade. Nonetheless, commonly used gene regions, including the SSU and ITS, do not enable reliable phylogenetic placement. Here, we present an improved database and pipeline for the phylogenetic determination of AMF using amplicons from the large subunit (LSU) rRNA gene. We improve our database and backbone tree by including additional outgroup sequences. We also improve an existing bioinformatics pipeline by aligning forward and reverse reads separately, using a universal alignment for all tree building, and implementing a BLAST screening prior to tree building to remove non-homologous sequences. Finally, we present a script to extract AMF belonging to 11 major families as well as an amplicon sequencing variant (ASV) version of our pipeline. We test the utility of the pipeline by testing the placement of known AMF, known non-AMF, and *Acaulospora* sp. spore sequences. This work represents the most comprehensive database and pipeline for phylogenetic placement of AMF LSU amplicon sequences within the Glomeromycota clade.

**Supplementary information:**

The online version contains supplementary material available at 10.1007/s00572-022-01068-3.

## Introduction

Arbuscular mycorrhizal fungi (AMF) are ubiquitous plant symbionts, associating with approximately 70% of plant species worldwide (Brundrett and Tedersoo 2018), yet difficulties associated with culturing and identifying these symbionts via traditional taxonomic methods have prevented a full assessment of their diversity and understanding of their ecology. AMF species differ in their impacts on plant growth (Hoeksema et al. 2018), plant defense (Bennett et al. 2009), and ecosystem functions (Bedini et al. 2009). Consequently, variation in AMF community composition alters terrestrial ecology (Van Der Heijden et al. 1998; Vogelsang et al. 2006; Koziol et al. 2018). Therefore, understanding patterns and dynamics of AMF composition is a research priority. Quantification of AMF species diversity and community composition has increasingly relied on metabarcoding of rRNA gene sequences from field samples (Öpik et al. 2014). However, to date, no single region of the rRNA gene has been universally accepted as optimal for AMF environmental sequencing.

Although it has been suggested that the internal transcribed spacer (ITS) region of the rRNA gene should be adopted as the universal fungal marker (Schoch et al. 2012; Lindahl et al. 2013), for the AMF in Glomeromycota this region is not optimal for two major reasons (Stockinger et al. 2010; Schoch et al. 2012). First, the sequence matching approach used for ITS sequences with other fungi is of limited utility for AMF because of the poor representation and poor curation of AMF sequences in ITS sequence databases (Bidartondo 2008; Stockinger et al. 2010). This database problem cannot be easily addressed because a high proportion of AMF encountered in environmental samples are undescribed. Therefore, this database matching approach results in a loss of most environmental sequences due to absence from databases and makes discovery of new taxa via sequencing impossible. Second, while phylogenetic approaches can be used to identify new sequences of AMF, this approach cannot be used for ITS amplicons because rapid sequence evolution of the ITS does not generate reliable trees (Nilsson et al. 2008). This renders the ITS ill-suited as a universal Glomeromycota marker in environmental sequencing.

An alternative region that commonly has been used for environmental sequencing of AMF is the small subunit, or SSU (Öpik et al. 2014). The use of this region is supported by a well-developed and curated database for AMF (Öpik et al. 2010; Davison et al. 2015) and the potential to build phylogenetic trees for clade placement (Franck et al. 2020). Nonetheless, the SSU region has the disadvantage of being slow-evolving and therefore not sufficiently variable to adequately resolve AMF species (Krüger et al. 2009; Bruns and Taylor 2016; Schlaeppi et al. 2016).

Recently, there is growing interest in sequencing the full length of the SSU, ITS, and LSU together (SSU-ITS-LSU) using long-read sequencing technology, such as PacBio (Tedersoo et al. 2018; Kolaříková et al. 2021). However, the implementation of phylogenetic placement using these technologies is not fully developed for AMF, particularly for community studies, and is still prohibitively costly for many. Further, although these long AMF reads may be used in phylogenetic placement of taxa, this will require increased user knowledge and computing resources due to differing rates of evolution across the SSU, ITS, and LSU regions. Therefore, although these long reads have the potential to lead to improved identification of AMF, the current standard is likely to remain Illumina amplicon sequencing.

A useful alternative to the ITS, SSU, or long reads is the large subunit (LSU). This region consistently shows utility for taxonomic resolution for AMF (Krüger et al. 2012; Hart et al. 2015; House et al. 2016; Delavaux et al. 2021), making it useful to access species diversity and community composition based on environmental AMF sequencing. This region is faster evolving than the SSU, but slower than the ITS, making it ideal for phylogenetic tree building and placement of unknown environmental sequences. Nonetheless, use of the LSU region for environmental sequencing of AMF is not common (Gollotte et al. 2004; Lekberg et al. 2013; House and Bever 2018; Vieira et al. 2018; Schütte et al. 2019), potentially due to bioinformatic challenges in implementation. To address this gap, we previously outlined the use of a newly created database and pipeline for the LSU region for placement in the Glomeromycota phylum (Delavaux et al. 2021). This curated database and the resulting backbone tree were built using sequences from Glomeromycota species across all major families for which published LSU sequences were available, and was supplemented by additional unpublished sequences from the International Culture Collection of (Vesicular) Arbuscular Mycorrhizal Fungi (INVAM). The important advance of this pipeline was the ability to place any environmental sequence into a well-supported reference tree to determine putative AMF, based on placement in the conserved AMF clade.

Subsequent implementation of our pipeline revealed that certain sequences placed into the tree appeared to be non-homologous with the LSU gene region. This was detected through the identification of abnormal clumping and long branch lengths assigned to study sequences. Although the cause of this issue remains uncertain, we incorporate a new step in the pipeline to address it. In the process, we also implement several improvements to both the database and pipeline to create the most up-to-date and complete version of the LSU AMF database and pipeline for placement of environmental sequences.

Here, we expand our reference database to include more fungal groups than in the previous version to aid in placement of environmental sequences, increase alignment accuracy in tree building by ensuring all reference sequences start and end at the same location, and aligning forward and reverse reads separately (Table 1). We also implement a BLAST screening prior to tree building to eliminate non-homologous reads. We further present code to extract operational taxonomic units (OTUs) falling within 11 major AMF families and present a second pipeline for amplicon sequencing variants (ASVs; 100% OTUs). Finally, we examine the utility of our pipeline by testing for correct placement of known AMF across the phylum, known Ascomycetes and Basidiomycetes (non-AMF) and *Acaulospora* sp. spore amplicon sequences. This work improves the utility of the database and pipeline, thereby making the LSU an attractive alternative for environmental sequencing of AMF.

**Table 1:** Pipeline steps and associated outputs

Step	Description	Major output
Initial processing	Primer (LROR/FLR2) and Illumina adaptor removal	Trimmed sequences
	Visualize quality to determine sequence length cutoff	Quality plots
Quality filter	DADA2 pipeline: filter, clean, and merge paired-end reads	ASV table and.fasta file
	Pre-screening BLAST against AMF reference database	BLAST filtered ASV table and.fasta file
	OTU clustering	BLAST filtered OTU table and.fasta file
Align sequences	Align reference and study sequences for forward (R1) and reverse (R2) reads	Forward and reverse.fasta alignments
	Concatenate forward and reverse alignments	Concatenated.fasta alignment
Tree building	Place study sequences (in batches) into backbone reference tree	RAxML trees
Extract AMF	Extract sequences in the Glomeromycota clade	Glomeromycota clade filtered OTU or ASV table and.fasta file
Extract AMF families	Extract sequences within each of the 11 Glomeromycota family clades	Family clade filtered OTU or ASV tables and.fasta files

## Methods and results

### Improved reference tree

#### Trimming reference sequences to pipeline primers

**Fig. 1:**
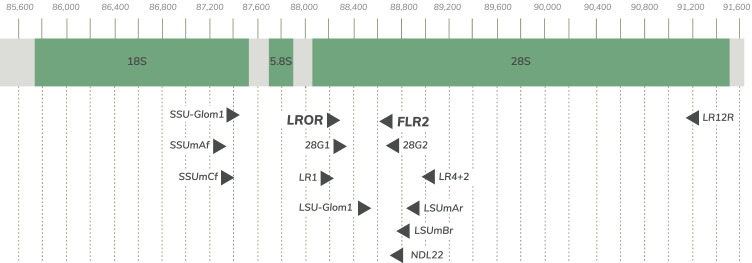
The rRNA gene region with commonly used AMF primers. Primers used in studies from which database sequences were extracted. The scale represents basepair number. The SSU is represented by 18S, while the LSU is represented by 28S. ITS1 and ITS2 are represented by the grey regions either side of 5.8S, with ITS1 to the left and ITS2 to the right. Full sequences can be found in Table S1

To ensure that our reference (database) sequences aligned optimally with study sequences (those sequences run through the pipeline) in our pipeline, all reference sequences were edited to begin and end at the primers with which the pipeline is built: LROR and FLR2. Many different primers were originally used to amplify reference sequences. INVAM sequences used the LR1 forward primer and the FLR2 reverse primer, while other sequences included used combinations of the LR1, 28G1, LR1, LROR, SSU-Glom1, SSUmAf, and SSUmCf forward primers and the 28G2, FLR2, LR12R, LR4 + 2, LSU-Glom1, LSUmAr, LSUmBr, and NDL22 reverse primers. To ensure that all sequences started and ended at our pipeline primers, we trimmed longer sequences to the LROR to FLR2 region using Cutadapt (Martin 2011). Because sequences that were originally amplified using the LR1, 28G1, 28G2, and LSU-Glom1 primers were slightly shorter than the region targeted by the LROR and FLR2 primers, IUPAC wildcard characters (“N”) were added to match the targeted region length. A list of these primers can be found in Table S1, while the position of all primers used in sequences included in the reference database within the rRNA gene can be found in Fig. 1.

#### Additional outgroup representation

**Fig. 2:**
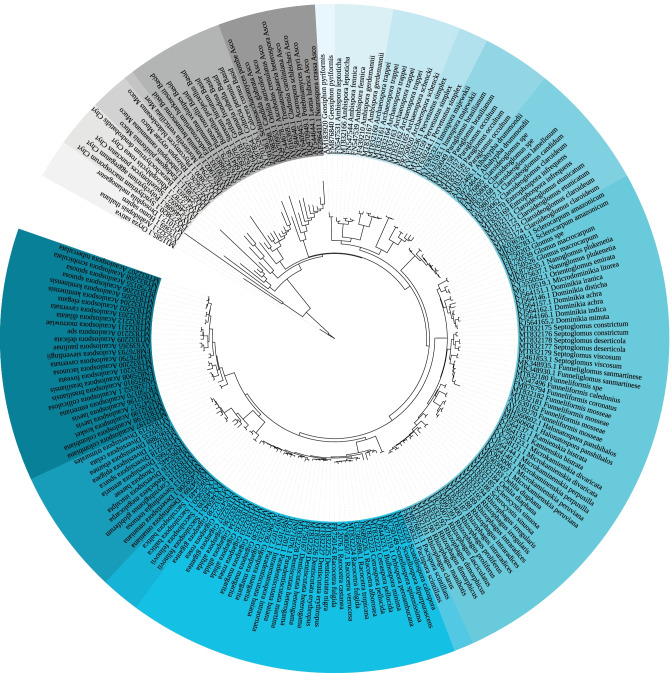
The new reference tree for placement of AMF via LSU amplicon sequences. This expanded tree includes 174 AMF across 11 families (indicated by shades of blue) and sequences representing the major neighboring clades of the Basidiomycota, Ascomycota, Mucoromycota, Mortierellomycota, and Chytridomycota as well as two animal and two plant outgroups (indicated by shades of grey)

To better discriminate between AMF and non-AMF taxa, we expanded the reference tree to include representative sequences from the major phyla neighboring the Glomeromycota including Chytridomycota, Mucoromycota, Mortierellomycota, Ascomycota, and Basidiomycota. Based on taxa from James et al. (2006), we initially built a large reference tree using all available LSU sequences of these non-AMF taxa along with our published database (Delavaux et al. 2021). For use in the pipeline, we then created a smaller tree with five sequences per clade for the Mucoromycota and Mortierellomycota (formerly the Zygomycota) and Chyrtidomycota and ten sequences per clade for Ascomycota and Basidiomycota to ensure efficient computation in the AMF LSU pipeline. In selecting these sequences, we chose the tips representing the edges of a given clade and one or two sequences from each subclade within that group. We further included two animals (*Homo sapiens*, *Drosophila melanogaster*) and two plants (*Arabidopsis thaliana*, *Oryza sativa*), using *Oryza sativa* as the new outgroup, based on the Interactive Tree of Life (Letunic and Bork 2019). Sequences were aligned using MAFFT (Katoh and Standley 2013) and a tree was constructed using RAxML v8 (Stamatakis 2014) with 1000 bootstrap replicates and the evolutionary model GTRGAMMA in QIIME2 (https://qiime2.org). This resulted in a well-supported new reference tree with 207 tips (Fig. 2).

### Improved pipeline

#### Pre-screening of representative sequences

**Fig. 3:**
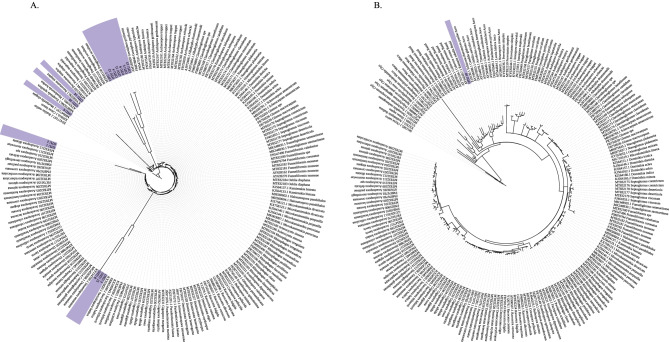
An example of non-homologous sequences corrected by the new pipeline. The same 15 OTUs were placed in the tree without an initial BLAST screening (**A**; shaded in purple), but including this step removed these non-homologous sequences, with only OTU 10 retained (**B**)

Early implementation of this pipeline revealed that certain taxa used in tree building appeared to be non-homologous with the LSU rRNA region, creating abnormal grouping of study sequences and unacceptably long-branch lengths (Fig. 3A). These sequences do not appear to be AMF and are not identifiable or had weak affiliations with prokaryotes based on a BLAST search. The cause of this issue is uncertain, but could be the result of Illumina biases, chimeras, or non-target amplification. Because sequences used to build trees must be homologous (as reflected in some basal level of similarity) with reference sequences to have a meaningful tree (e.g., must be LSU), we implemented a low-percent BLAST screening as a pre-tree-building screening step. Specifically, we retain all taxa in the cleaned sequences that show any similarity to the reference AMF sequences in the database. This ensures that sequences used in tree construction show homology with the AMF reference sequences and result in reliable placement (Fig. 3B). We used the same test dataset used in Delavaux et al. (2021), generated from soil samples collected from a midwestern tallgrass prairie system, to compare this pre-screening approach to our initial results. When comparing a sample of 15 sequences from the test dataset, we find that out of the 15 OTUs originally placed in a tree in the original version of the pipeline (Fig. 3A), only one OTU remains when implementing the BLAST pre-screening approach (Fig. 3B). Importantly, the clustering and long-branch lengths seen with our original pipeline are no longer detectable in the updated pipeline.

#### Improved alignment of study sequences

With the goal of improving alignment, we implemented two major changes. First, the pipeline now cuts reference sequences to the length of study sequences to mimic the Illumina reads generated using our primers (previously, reference sequences were kept full length). This takes the R1 (forward) and R2 (reverse) cutoff for the quality control of the study sequences and applies it to the reference database. Second, the R1 and R2 sections of the database and study sequences are each aligned separately in a universal alignment and subsequently concatenated for tree building. The R1 and R2 sections represent two separate sections of the LSU; therefore, we align them separately to remove any possibility of sections of the R1 of one sequence aligning with the R2 of another sequence. These changes result in a more reliable alignment than previously between reference and study sequences by (1) comparing only regions with data present in both study and reference sequences and (2) ensuring that the alignment does not incorrectly align the R1 with R2 regions.

#### Extracting glomeromycota families

We provide an additional script to extract family level taxa based on placement in the family clade based on our reference tree. This delineates 11 described families in the Glomeromycota: *Acaulosporaceae*, *Ambisporaceae*, *Archaeosporaceae*, *Claroideoglomeraceae*, *Diversisporaceae*, *Gigasporaceae*, *Glomeraceae*, *Pacisporaceae*, *Paraglomeraceae*, *Pervestustaceae*, and *Sacculosporaceae* (Fig. 2). Species used to delineate these families can be found in Table S2. We assessed the functioning of this pipeline component by testing the accurate placement of Illumina sequences from *Acaulospora* sp. spore DNA extractions (Table S3). We found that although only 25% of AMF OTUs were placed in the *Acaulosporaceae*, the majority (68.6%) of all AMF reads were placed in this family (Table S4). While a low fraction of OTUs were placed in the *Acaulosporaceae*, this low number of OTUs comprises more sequences on average than the remaining 75% of OTUs. This confirms that this family clade placement method is working adequately, at least for the *Acaulosporaceae*. The OTUs and reads that were not placed in this family could either represent taxa that fall just outside the current *Acaulosporaceae* clade delimitation or may be amplifications of relic environmental DNA from the sterilized background soil used in culturing these spores.

#### Placement of known taxa using the pipeline

To confirm accurate placement of known taxa into our tree, we tested placement of sequences of known AMF and non-AMF into our backbone tree. We used 145 AMF sequences from Krüger et al. (2009) and 10 Ascomycota and 10 Basidiomycota from James et al. (2006). Sequences were trimmed to LROR/FLR2 primers and then cut to 150 bp for the forward and reverse reads and joined using the Biostrings (Pages et al. 2016) and stringr (Wickham and Wickham 2019) packages in R. We selected 150 bp as the cutoff as this is a conservative quality cutoff for Illumina sequences. Using our pipeline, we found that 100% of AMF sequences were accurately placed in the AMF clade, while 100% of non-AMF were placed outside of the AMF clade. This result shows that our pipeline is performing very well, at least with these previously described taxa, placing all known AMF inside, and all known non-AMF outside, of the Glomeromycota phylum.

#### The value of phylogenetic placement of AMF

We again used the test dataset to evaluate the reliability of our placement into the Glomeromycota clade as well as the utility of phylogenetic placement over BLAST. We conducted BLASTn (blast.ncbi.nlm.nih.gov) on all phylogenetically placed Glomeromycota OTUs. Overall, we found that within the proportion of AMF OTUs from our pipeline, most were confirmed by BLAST as Glomeromycota (R1: 91.0%, R2: 92.2%; average: 91.6%). However, we confirm the utility of phylogenetic placement as there were substantial BLAST hits labelled as fungus, oomycetes, or without a BLAST hit (R1: 9.1%, R2: 7.8%; average: 8.45%).

#### ASV pipeline

We adapted our pipeline to process amplicon sequence variants (ASVs). To accomplish this, we omit the portion of the pipeline that creates OTUs. Because the pipeline generates ASVs that are then clustered into OTUs, we simply removed the final clustering to retain ASVs. Historically, studies looking at AMF used the OTU approach because (1) individual AMF spores show high levels of polymorphism, which are reduced when aggregated at the OTU level (House et al. 2016; Montoliu-Nerin et al. 2021); (2) sufficiently controlling for sequencing errors at the ASV resolution still posed a challenge; and (3) accommodating the sheer volume of data was prohibitive. Although AMF polymorphism may be reduced at the OTU level, this issue is present for both OTUs and ASVs (House et al. 2016; Montoliu-Nerin et al. 2021). Recent methods sufficiently control for sequencing errors, allowing for the use of ASVs (Callahan et al. 2016). In addition, our pre-tree building BLAST screening substantially reduces the number of taxa used in subsequent tree building, enabling the use of ASVs.

## Discussion

Here, we present an updated and improved database and pipeline for the phylogenetic placement of AMF amplicon environmental sequences using the LSU region of the rRNA gene, building on the initial database and pipeline provided by Delavaux et al. (2021). We have tested and confirmed the efficacy of this pipeline using known AMF, outgroup representatives, and spore-based sequences from *Acaulospora* sp. This improved database and pipeline improves our ability to identify environmental amplicon sequences as putative AMF and putative members of AMF family clades.

In addition, we have increased the utility of this pipeline by providing ASV as well as OTU adapted versions and by including code to extract clades at the family level. There are two major advantages to working with ASVs: increased resolution of data and possibility to compare across studies (Callahan et al. 2017). Because OTUs are created based on similarity within a given set of study sequences, they cannot be compared across studies. To compare across studies would require all raw sequences from respective studies to be reanalyzed together from the OTU clustering step forward. ASVs, however, represent unique sequence variants found in the data, so these will be comparable across studies. OTUs are typically grouped at a given percent similarity across all sequences, even though certain taxa may show higher or lower percent similarity (House et al. 2016), resulting in differential levels of resolution across the phylum. Use of ASVs essentially groups OTUs at the highest level of similarity (100%), resulting in unique sequences observed in the data. Therefore, ASVs may be preferable to identify all unique sequences and avoid making assumptions about the similarity threshold to apply across the Glomeromycota. Ultimately, the decision to use OTUs or ASVs will depend on study goals and available computing resources.

Together, these tools will be especially powerful in environmental sequencing of little studied systems, such as in the tropics or boreal forests. In our test dataset, we found that approximately 8% of phylogenetically determined putative AMF do not match to AMF sequences registered in NCBI (BLAST). This percentage of OTUs that were phylogenetically placed by our pipeline in the Glomeromycota but not identified as such by BLAST are conservative, given that the test data are from the historically highly sampled tallgrass prairie biome (Bennett and Classen 2020). Therefore, other less sampled areas should result in a greater proportion of previously unidentified putative AMF via this pipeline, presenting an important advance to identifying hitherto undescribed AMF. This appreciable amount of data would typically be discarded but may well represent new fungal taxa, and would help provide a full picture of AMF diversity locally and globally.

With additional full-length LSU sequences, the reference tree will be expanded further, incorporating potential new clades within the Glomeromycota presently not represented. Although we found a very high success rate of placement of AMF with known AMF sequences, these sequences tend to be confined to known and well-studied families. With an expanded set of AMF taxa in the reference tree, including sequences from INVAM voucher specimens, we may expand the precision of AMF placement to additional families and clades. In addition, a dense reference tree may eventually be used to identify species with sufficient sequences. In its present form, this tool helps researchers identify putative AMF as well as putative AMF belonging to the major 11 families, representing an important advance in identifying the unculturable diversity of the Glomeromycota.

## Supplementary information

Below is the link to the electronic supplementary material.

Supplementary file1 (DOCX 19 KB)

## Data Availability

The test dataset and spore sequences analyzed in the study are available in the NCBI repository, Project #PRJNA648993 and #PRJNA752715, respectively. All other sequences used for test placement can be found in cited references in the text. For sequences included in the reference tree, see respective accession numbers in tip names.

## Code availability

All code can be found at https://github.com/c383d893/AMF-LSU-Database-and-Pipeline2.
