# Supplementary material for: Environmental identification of arbuscular mycorrhizal fungi using the LSU rDNA gene region: an expanded database and improved pipeline
Source: Mycorrhiza. 2022 Jan 31;32(2):145–53. doi: 10.1007/s00572-022-01068-3 (PMC8907093; doi:10.1007/s00572-022-01068-3)
Supplement: Supplementary file 1 — Supplementary file1 (DOCX 19 KB) [file 572_2022_1068_MOESM1_ESM.docx]

**Supplementary information**

**Table S1.** The rRNA gene region AMF primers

Forward and reverse primers used in studies from which database sequences were extracted. Primers for which the pipeline is developed are marked with an asterisk (*).

| Primer Name | Primer Sequence (5' -3') |
| --- | --- |
| ***Forward*** | |
| LROR* | ACCCGCTGAACTTAAGC |
| LR1 | GCATATCAATAAGCGGAGGA |
| 28G1 | CATGGAGGGTGAGAATCCCG |
| SSU-Glom1 | ATTACGTCCCTGCCCTTTGTACA |
| SSUmAf1 | TGGGTAATCTTTTGAAACTTYA |
| SSUmAf2 | TGGGTAATCTTRTGAAACTTCA |
| SSUmCf1 | TCGCTCTTCAACGAGGAATC |
| SSUmCf2 | TATTGTTCTTCAACGAGGAATC |
| SSUmCf3 | TATTGCTCTTNAACGAGGAATC |
| *Reverse* | |
| FLR2* | GACGTAATGGCTTTAAACGA |
| LSU-Glom1 | TGAAAGGGAAACGATTGAAG |
| NDL22 | CGTCTTGAAACACGGACCA |
| LR4+2 | AGCCAGAGGAAACTCTGGT |
| 28G2 | CGTTAAGGATGTTGACGTAATGG |
| LR12R | CTGAACGCCTCTAAGTCAGAA |
| LSUmAr1 | TTTGATAGATTTGAGTGTGAGC |
| LSUmAr2 | ATCGATAGAATTGAGTTAGAGC |
| LSUmAr3 | TTTGATAGATTTGAGTAAGAGCA |
| LSUmAr4 | TCGATAGGTTTGAGTAAGAGC |
| LSUmBr 1 | TCTAACATATATGCGAGTGTTH |
| LSUmBr 2 | TCTAACATGTGTGCGAGTGTT |
| LSUmBr 3 | TCTAACATGTATGCGAGTGTT |
| LSUmBr 4 | TCTAACATATGTGCGAGTGTTT |
| LSUmBr 5 | TCTAGCATATATGCGAGTGTT |

**Table S2.** Taxa used to delineate families in our pipeline

Taxa used to extract eleven AMF families in the pipeline.

| **Family** | **Taxa edge 1** | **Taxa edge 2** |
| --- | --- | --- |
| Acaulosporaceae | MT832197 Acaulospora colombiana | MT832207 Acaulospora tuberculata |
| Ambisporaceae | FN547531 Ambispora leptoticha | MT832167 Ambispora gerdemannii |
| Archaeosporaceae | MT832160_Archaeospora_trappei | FR750020 Archaeospora schencki |
| Claroideoglomeraceae | AJ972466.1 Albahypha drummondii | MT832174 Claroideoglomus claroideum |
| Diversisporaceae | KF154769.1 Desertispora omaniana | MT832219_Diversispora_trimurales |
| Gigasporaceae | MT832220_Scutellospora_calospora | MT832238_Gigaspora_gigantea |
| Glomeraceae | MK036781.1 Sclerocarpum amazonicum | MT832191 Rhizophagus clarus |
| Pacisporaceae | FM876831.1_Pacispora_scintillans | FM876832.1_Pacispora_scintillans |
| Paraglomeraceae | KY630232.1_Innospora_majewski | MT832155_Paraglomus_occultum |
| Pervestustaceae | KY630236.1_Pervetustus_simplex | KY630244.1_Pervetustus_simplex |
| Sacculosporaceae | FR865449.1 Sacculospora baltica | KX345943.1 Sacculospora felinovii |

**Table S3.** *Acaulospora* sp. isolates sequenced

Information about each isolate species, INVAM code and country of origin.

| **Species** | **INVAM Code** | **Country** |
| --- | --- | --- |
| *Acaulospora mellea* | AU959N | Australia |
| *Acaulospora mellea* | MA461B | USA |
| *Acaulospora mellea* | MN212 | USA |
| *Acaulospora mellea* | KS304 | USA |
| *Acaulospora mellea* | JA114 | USA |
| *Acaulospora mellea* | JA401B | Japan |
| *Acaulospora dilatata* | WV204 | USA |
| *Acaulospora morrowiae* | AK101B | USA |
| *Acaulospora morrowiae* | CU141 | Cuba |
| *Acaulospora morrowiae* | EY104 | Egypt |
| *Acaulospora morrowiae* | FL208N | USA |
| *Acaulospora morrowiae* | HA725A | USA |
| *Acaulospora morrowiae* | SY157 | Syria |
| *Acaulospora morrowiae* | KR102 | South Korea |
| *Acaulospora delicate* | AZ661 | USA |
| *Acaulospora rugosa* | WV949 | USA |
| *Acaulospora lacunose* | WV613 | USA |
| *Acaulospora colombiana* | CL356 | Colombia |
| *Acaulospora* sp. | TU102C | Turkey |

**Table S4.** *Acaulospora* sp. spore sequence placement test

*In silico* test based on AMF sequences from *Acaulospora* sp. spores confirmed adequate accuracy in reads correctly placed. While a low fraction of OTUs are placed in the *Acaulosporaceae (25%)*, this low number of OTUs are made up of more sequences on average than the remaining 75% of OTUs, as they contain 69% of sequence reads.

|  | **Reads (#)** | **Reads (%)** | **OTUs (#)** | **OTUs (%)** |
| --- | --- | --- | --- | --- |
| Placed in known family within Glomeromycota | 646148 |  | 136 |  |
| Acaulosporaceae | 624047 | 68.5658 | 34 | 25.000 |
| Ambisporaceae | 0 | 0.0000 | 0 | 0.0000 |
| Archaeosporaceae | 0 | 0.0000 | 0 | 0.0000 |
| Claroideoglomeraceae | 8571 | 0.9417 | 6 | 4.4118 |
| Diversisporaceae | 11 | 0.0012 | 1 | 0.7353 |
| Gigasporaceae | 0 | 0.0000 | 0 | 0.0000 |
| Glomeraceae | 13396 | 1.4719 | 30 | 22.0588 |
| Pacisporaceae | 0 | 0.0000 | 0 | 0.0000 |
| Paraglomeraceae | 123 | 0.0135 | 1 | 0.7353 |
| Sacculosporaceae | 0 | 0.0000 | 0 | 0.0000 |
| Not placed in known family within Glomeromycota | 263995 | 29.0059 | 64 | 47.0588 |
